# Complete genome sequence of *Geobacillus icigianus* ID-2 from an Indonesian hot spring

**DOI:** 10.1128/mra.00621-25

**Published:** 2025-07-31

**Authors:** Jae-Yoon Sung, Mun-Hoe Lee, Seong-Bo Kim, Sang-Jae Lee, Dong-Woo Lee

**Affiliations:** 1 Department of Biotechnology, Yonsei University, Seoul, South Korea; 2 Department of Integrative Biotechnology, Yonsei University, Seoul, South Korea; 3 Bio-Living Engineering Major, Global Leaders College, Yonsei University, Seoul, South Korea; 4 Department of Bioscience and Research Center for Extremophiles and Marine Microbiology, Silla University, Busan, South Korea; DOE Joint Genome Institute, Berkeley, California, USA

**Keywords:** *Geobacillus icigianus*, thermophile, genome, lipase, hot spring

## Abstract

We report the complete genome of lipolytic *Geobacillus icigianus* strain ID-2, isolated from an Indonesian hot spring. The genome encodes 3,816 genes and exhibits four distinct methylation patterns. These data expand our understanding of *Geobacillus* species and provide a foundation for their use as microbial bioengineering platforms.

## ANNOUNCEMENT

*Geobacillus spp*. are known for their robust enzyme activity and thermostability, making them attractive for industrial applications (1–4). We sequenced the genome of *Geobacillus icigianus* strain ID-2, which was isolated from a geothermal hot spring water sample at Tangkuban Perahu, Java Island, Indonesia (6°45′42″S, 107°37′4″E) in October 1999. A 200 µl aliquot of serially diluted water sample was cultured on EM-1 agar plates (containing 0.1% (w/v) yeast extract, 5 mL of olive oil, 2 g of NaCl, 0.4 g of MgSO_4_⋅7H_2_O, 0.7 g of MgCl_2_⋅6H_2_O, 0.5 g of CaCl_2_⋅2H_2_O, 0.3 g of KH_2_PO_4_, 0.3 g of K_2_HPO_4_, 0.5 g of (NH_4_)_2_SO_4_, 1 mL of Wolin’s vitamin solution, and 1.0 mL of Wolin’s trace element solution (DSM cat. no. 141)) at 60°C for 5 days to investigate oil-degrading capabilities and biotechnological potential (5). A single colony was selected and further purified through successive streaking.

Genomic DNA was extracted using the Wizard Genomic DNA Purification Kit (Promega, USA) and used for both long-read and short-read sequencing. For long reads generation, DNA was sheared using a g-TUBE (Covaris Inc., Woburn, MA, USA) and size-selected for fragments <17 kb using AMPurePB magnetic beads (Beckman Coulter Inc., CA, USA). Libraries were constructed using the (3.0) PacBio Microbial Library Prep Kit (Pacific Biosciences, CA, USA) and sequenced on a PacBio Revio system, yielding 513,459 raw HiFi Reads (3,996,815,665 bp) with an average length of 7,784 bp (N_50_ length, 9,051 bp). The hierarchical genome-assembly process v4 generated a 3,803,328 bp circular chromosome with 51.5% GC content at 1051.1× coverage (Table 1). Taxonomic identification of *G. icigianus* ID-2 was determined based on an Average Nucleotide Identity (ANI) value of 98.0% compared to the reference strain *G. icigianus* G1w1 (GCA_000750005.2) using the NCBI Prokaryotic Genome Annotation Pipeline (PGAP).

**TABLE 1:** Genomic features of *G. icigianus* strain ID-2^*a*^

Feature	ID-2
Genome size (bp)	3,803,328
No. of contigs	1
GC content (%)	52.0
Total number of genes	3,816
Protein coding genes (CDS)	3,463
rRNA genes (5S, 16S, and 23S)	26 (8, 9, and 9)
tRNA genes	89
ncRNA	5
Pseudogenes	233
CRISPR arrays	5
GenBank Accession	CP187452

^*a*^ CDS, Coding sequence.

Short-read Illumina sequencing was performed for error correction using the TruSeq Nano DNA Prep Kit. Paired-end libraries (2 × 150 bp) were sequenced on a NovaSeq 6000 (Illumina), yielding 13,306,306 reads (2,009,252,206 bp). Quality control and adapter trimming were performed using Trimmomatic v0.39 (6) and FastQC v0.11.9 (7). Variant calling was performed using bcftools with parameters “--minDP 5 --minQ 30” (8), and the consensus genome was polished accordingly.

Genome annotation was performed using the NCBI PGAP v6.10 (9). Methylation motif analysis incorporated in SMRTlink v13.1 revealed a type III R-M system (RM.*GicID2*IV; GCC^6m^AT), a type II R-M system (RM.*GicID2*III; CTGC^6m^AG, an isoschizomer of PstI), and two type I R-M systems (RSM.*GicID2*II; G^6m^ATNNNNNNRTTC/GA^6m^AYNNNNNNATC), (RSM.*GicID2*I; AC^6m^ANNNNNNRTCG/CG^6m^AYNNNNNNTGT) (https://rebase.neb.com/cgi-bin/pacbioget?91916). All tools were run with default parameters unless otherwise specified.

Strain ID-2 also exhibited C4 and C8 esterase/lipase activity potential (XRN34578.1), consistent with activity observed in *Geobacillus thermodenitrificans* ID-1 (formerly *Bacillus thermoleovorans* ID-1) isolated from the same site. Moreover, ID-2 carries CRISPR systems Type I-B, Type II-C, and two copies of Type III-B, indicating its potential in genome editing and enzyme biocatalysis (10, 11). These genomic features underscore ID-2’s adaptability and utility for lipid hydrolysis and industrial enzyme production.

## Data Availability

The genome sequence of *G. icigianus* ID-2 is available in GenBank under accession number CP187452.1 (BioProject: PRJNA1249976). Raw sequencing data are available under SRA accession numbers SRX28376586 and SRX28608945 (BioSample: SAMN48008355). Methylation motifs are available under Supplementary Files accession number SUPPF_0000005667.
